# Social determinants of health and clinical outcomes among patients with atrial fibrillation: evidence from a global federated health research network

**DOI:** 10.1093/qjmed/hcad275

**Published:** 2023-12-07

**Authors:** A H Simoni, T Bucci, G F Romiti, J Frydenlund, S P Johnsen, A H Abdul-Rahim, G Y H Lip

**Affiliations:** Liverpool Centre for Cardiovascular Science at University of Liverpool, Liverpool John Moores University and Liverpool Heart & Chest Hospital, Liverpool, UK; Department of Clinical Medicine, Danish Center for Health Services Research, Aalborg University, Aalborg, Denmark; Liverpool Centre for Cardiovascular Science at University of Liverpool, Liverpool John Moores University and Liverpool Heart & Chest Hospital, Liverpool, UK; Department of General and Specialized Surgery, Sapienza University of Rome, Rome, Italy; Liverpool Centre for Cardiovascular Science at University of Liverpool, Liverpool John Moores University and Liverpool Heart & Chest Hospital, Liverpool, UK; Department of Translational and Precision Medicine, Sapienza University of Rome, Rome, Italy; Liverpool Centre for Cardiovascular Science at University of Liverpool, Liverpool John Moores University and Liverpool Heart & Chest Hospital, Liverpool, UK; Department of Clinical Medicine, Danish Center for Health Services Research, Aalborg University, Aalborg, Denmark; Department of Clinical Medicine, Danish Center for Health Services Research, Aalborg University, Aalborg, Denmark; Liverpool Centre for Cardiovascular Science at University of Liverpool, Liverpool John Moores University and Liverpool Heart & Chest Hospital, Liverpool, UK; Stroke Division, Department of Medicine for Older People, Whiston Hospital, St Helens and Knowsley Teaching Hospitals NHS Trust, Prescot, UK; Liverpool Centre for Cardiovascular Science at University of Liverpool, Liverpool John Moores University and Liverpool Heart & Chest Hospital, Liverpool, UK; Department of Clinical Medicine, Danish Center for Health Services Research, Aalborg University, Aalborg, Denmark

## Abstract

**Background:**

Few studies have investigated the role of social determinants of health (SDoH) in patients with atrial fibrillation (AF).

**Aim:**

To investigate the relationship between SDoH and adverse events in a large multinational AF cohort.

**Design:**

Retrospective study utilizing a global federated health research network (TriNetX).

**Methods:**

Patients with AF were categorized as socially deprived defined according to ICD codes based on three SDoHs: (i) extreme poverty; (ii) unemployment; and/or (iii) problems related with living alone. The outcomes were the 5-year risk of a composite outcomes of all-cause death, hospitalization, ischemic heart disease (IHD), stroke, heart failure (HF) or severe ventricular arrhythmias. Cox regression was used to compute hazard rate ratios (HRs) and 95% confidence intervals (CIs) following 1:1 propensity score matching (PSM).

**Results:**

The study included 24 631 socially deprived (68.8 ± 16.0 years; females 51.8%) and 2 462 092 non-deprived AF patients (75.5 ± 13.1 years; females 43.8%). Before PSM, socially deprived patients had a higher risk of the composite outcome (HR 1.9, 95% CI 1.87–1.93), all-cause death (HR 1.34, 95% CI 1.28–1.39), hospitalization (HR 2.01, 95% CI 1.98–2.04), IHD (HR 1.67, 95% CI 1.64–1.70), stroke (HR 2.60, 95% CI 2.51–2.64), HF (HR 1.91, 95% CI 1.86–1.96) and severe ventricular arrhythmias (HR 1.83, 95% CI 1.76–1.90) compared to non-deprived AF patients. The PSM-based hazard ratios for the primary composite outcome were 1.54 (95% CI 1.49–1.60) for the unemployed AF patients; 1.39 (95% CI 1.31–1.47) for patients with extreme poverty or with low income; and 1.42 (95% CI 1.37–1.47) for those with problems related with living alone.

**Conclusions:**

In patients with AF, social deprivation is associated with an increased risk of death and adverse cardiac events. The presence of possible unmeasured bias associated with the retrospective design requires confirmation in future prospective studies.

## Introduction

Atrial fibrillation (AF) is the most common cardiac arrhythmia worldwide and is associated with poor clinical outcomes, including higher mortality and morbidity.^1–3^ More than 33 million people are diagnosed with AF worldwide, and the prevalence is expected to increase with the demographic changes of the general population since the risk of AF is strongly related to age.^1^^,^^4^^,^^5^ Patients with AF are at high risk for death, ischemic heart disease (IHD), heart failure (HF) and other cardiovascular events.^1^^,^^3^^,^^5–8^

In the last decades, growing evidence suggests that the risk of adverse events in AF patients, behind the presence of classical cardiovascular risk factors, is also related to the social determinants of health (SDoH).^1^^,^^9–11^ The latter, including occupation, cohabitation, income level and poverty are known to represent individual pathways for the disparity in severe health outcomes^12–15^ and some studies suggest that SDoH affects the risk of poorer clinical outcomes including IHD, HF, stroke and mortality and in patients with AF.^1^^,^^3^^,^^5–7^ Despite substantial progress being made in improving health, social disparities between population groups have persisted and remain marked and prominent, especially within cardiovascular diseases and mortality.^5^^,^^15^^,^^16^ SDoH may affect the clinical outcomes after AF through multiple pathways, including influencing the ability to change to a healthier lifestyle behavior, persistence to prescribed medications, and undertreatment and underuse of health services among patients with AF who are unemployed, have low income, live in poverty or live alone.^5^^,^^15^

This study aimed to investigate the association between SDoH and clinical outcomes, including all-cause death, hospitalization, IHD, stroke, HF and severe ventricular arrhythmias, in a large AF population from a global federated health research network. The SDoH domains investigated included extreme poverty or low income, unemployment and problems related to living alone.

## Methods

This was a retrospective observational study conducted within TriNetX, a global federated health research network with access to electronic medical records (EMRs) from participating healthcare organizations including academic medical centers, specialty physician practices and community hospitals covering ∼69.8 million individuals, mainly in the USA. Within this network, available data include demographics, diagnoses using International Classification of Diseases, Ninth Revision and Tenth Revision, Clinical Modification (ICD-10-CM) codes and medications. More information can be found online (https://trinetx.com/company-overview/).

### Cohort

The research on the TriNetX online research platform was performed on 26 November 2022 for patients aged ≥18 years with AF or flutter (ICD-10-CM code I48). As previously described,^17^ the baseline index event was the first AF diagnosis reported into the TriNetX platform. Any medical diagnosis and cardiovascular procedures or treatment registered into the system within 5 years from the index event were considered as an individual baseline characteristic. At the time of the construction of the cohort, 64 participating healthcare organizations had data available for patients who met the characteristics of interest for the study.

### Exposure

The patients were categorized as socially deprived or non-deprived based on presentation of three characteristics: (i) extreme poverty (ICD-10-CM Z59.5) and or with low income (ICD-10-CM Z59.6); (ii) unemployment (ICD-10-CM Z56.0); and (iii) problems related with living alone (ICD-10.CM Z 60.2).^18^^,^^19^ Hence, the patients with AF were categorized as socially deprived if they presented any of these characteristics, and patients without any of these characteristics were categorized as non-deprived. Furthermore, the presentation of the three SDoH characteristics was evaluated individually.

### Outcomes

The primary outcome was the occurrence of a composite of major adverse cardiac events including all-cause death, stroke, IHD, HF, severe ventricular arrhythmias and hospitalization within 5 years after AF diagnosis. The choice of the components of this composite outcome was done to confirm in AF patients, the findings from previous studies that showed as SDoH are independent risk factors for such events in the general population.^20–22^ To better understand the specific weight associated with each indicator of SDoH, the secondary outcomes included each component of the composite outcome within 5 years after the index event. More detailed information about the ICD-10-CM codes utilized to identify the primary and secondary outcomes can be found in the Supplementary Table S1.

### Statistical analysis

All statistical analyses were performed on the TriNetX online platform. Continuous variables were expressed as mean (±standard deviation [SD]) and compared by *t*-test for independent variables, while categorical variables were expressed as counts and percentages and compared by chi-squared test. We performed a balanced 1:1 propensity score matching (PSM) to create balanced cohorts. For the PSM, the following variables were considered: age, sex, ethnicity, comorbidities (hypertension, HF, IHD, cerebrovascular disease, diabetes mellitus, overweight/obesity, chronic kidney disease, neoplasms, dyslipidemia, pulmonary hypertension, chronic rheumatic heart disease and chronic lower respiratory diseases), procedures (cardiac catheterization, echocardiography, electrocardiogram, cardiovascular monitoring services and intracardiac electrophysiological studies) and cardiovascular medications (beta-blockers, diuretics, lipid-lowering agents, angiotensin-converting enzyme inhibitors, calcium channel blockers, antianginals, anticoagulants and platelet aggregation inhibitors). Diagnosis codes are available in the Supplementary Table S2. Standardized mean differences (Std. diff.) were used to show the distribution of demographic and clinical data among the groups and calculated as the difference in the means or proportions of a particular variable divided by the pooled estimate of SD for that variable. After PSM, any baseline characteristic with an Std. diff. <0.1 was considered well matched. We used Cox proportional hazard regression analysis to investigate the 5-year risk for the primary and secondary outcomes both before and after the PSM. The risk was estimated by hazard rate ratio (HR) with 95% confidence intervals (CIs). For the primary outcome between *socially deprived* and *non-socially deprived* AF patients, we also reported Kaplan–Meier curves, and survival distribution was compared with log-rank test. For the survival analysis, a two-sided *P* values <0.05 was considered statistically significant. All the analyses were performed on the TriNetX platform using R software v3.3.

### Data availability and ethical approval

TriNetx is a research network utilized for several scientific purposes, compliant with the Health Insurance Portability and Accountability Act and the US federal law which protects the privacy and security of healthcare data, including de-identified data as per the de-identification standard of the HIPAA Privacy Rule (https://trinetx.com/real-world-resources/publications/). To gain access to the data in the TriNetX research network, requests are directed to TriNetX and a data sharing agreement is required. As a federated research network, studies using the TriNetX health research network do not need ethical approval as no patient identifiable identification is received. Further information about the data extraction from TriNetX is reported in the Supplementary Material.

## Results

### Baseline characteristics before propensity score matching

The initial cohorts consisted of 25 711 socially deprived patients with AF and 2 462 092 non-deprived patients with AF. The socially deprived cohort of patients with AF was composed of 10 510 (41%) unemployed, 3811 (14.7%) with extreme poverty or with a low income and 11 390 (44.3%) with problems related to living alone. A baseline comparison of the characteristics of patients with AF classified as socially deprived or non-deprived is shown in Table 1. The socially deprived patients were younger, more often female, Black or African American, and with a higher prevalence of cardiovascular risk factors than the non-deprived AF patients.

**Table 1.** Baseline characteristics of patients with atrial fibrillation

	Before propensity score match			After propensity score match		
	Socially deprived (*n* = 24 631)	Non-socially deprived (*n* = 2 462 092)	Std. diff.	Socially deprived (*n* = 24 627)	Non-socially deprived (*n* = 24 627)	Std. diff.
Age, years (±SD)	68.8 ± 16.0	75.5 ± 13.1	0.463	68.8 ± 15.9	69.1 ± 16.0	0.022
Female	12 760 (51.8)	1 079 576 (43.8)	0.160	12 756 (51.8)	13 169 (53.5)	0.034
White	17 837 (72.4)	1 878 987 (76.3)	0.089	17 835 (72.4)	17 755 (72.1)	0.007
Black or African American	3945 (16.0)	227 991 (9.3)	0.204	3943 (16.0)	4051 (16.4)	0.012
Hypertension	13 636 (55.4)	806 136 (12.9)	0.468	13 632 (55.4)	13 435 (54.6)	0.016
Obesity	6446 (26.2)	210 325 (8.5)	0.479	6446 (26.2)	6637 (27.0)	0.018
Diabetes mellitus	7675 (31.2)	354 729 (14.4)	0.408	7672 (31.2)	7700 (31.3)	0.002
Chronic kidney disease	4640 (18.8)	208 390 (8.5)	0.306	4638 (18.8)	4622 (18.8)	0.002
Pulmonary hypertension	2956 (40.0)	604 055 (24.5)	0.281	2953 (12.0)	3034 (12.3)	0.010
Ischemic heart disease	7617 (30.9)	417 021 (16.9)	0.332	7613 (24.2)	7457 (30.3)	0.014
Heart failure	5465 (22.2)	256 172 (10.4)	0.323	5463 (22.2)	5470 (22.2)	0.001
Cerebrovascular diseases	4978 (20.2)	220 919 (9.0)	0.322	4975 (20.2)	4965 (20.2)	0.001
Chronic lower respiratory diseases	6847 (27.8%)	282 923 (11.5%)	0.419	6843 (27.8)	6965 (28.3)	0.011
Neoplasms	8257 (33.5%)	422 349 (17.2%)	0.383	8253 (33.5)	8281 (33.6)	0.002
Cardiac catheterization procedures	2214 (9.0)	104 810 (4.3)	0.191	2213 (9.0)	2202 (8.9)	0.002
Echocardiography procedures	8007 (32.5)	408 553 (16.6)	0.376	8003 (32.5)	7914 (32.1)	0.008
Electrocardiogram, routine ECG with at least 12 leads	12 608 (51.2)	650 076 (26.4)	0.526	12 604 (51.2)	12 476 (50.7)	0.008
Cardiovascular monitoring services	1481 (6.0)	74 781 (3.0)	0.144	1480 (6.0)	1441 (5.9)	0.007
Intracardiac electrophysiological procedures/studies	376 (1.5)	20 555 (0.8)	0.064	376 (1.5)	378 (1.5)	0.001
Lipid-lowering drugs	9060 (42.5)	672 266 (27.3)	0.204	9059 (36.8)	8693 (35.3)	0.031
Beta-blockers	10 474 (47.6)	743 364 (30.2)	0.258	10 471 (42.5)	10 159 (41.3)	0.026
Diuretics	9330 (37.9)	606 735 (24.6)	0.288	9328 (37.9)	9176 (37.3)	0.013
Calcium channel blockers	6829 (27.7)	444 972 (18.1)	0.231	6826 (27.7)	6682 (27.1)	0.013
ACE inhibitors	6938 (25.4)	397 944 (16.2)	0.292	6936 (28.2)	6843 (27.8)	0.008
Angiotensin II inhibitors	3351 (13.6)	259 172 (10.5)	0.095	3351 (13.6)	3237 (13.1)	0.014
Anticoagulants	10 915 (44.3)	664 504 (27.0)	0.368	10 911 (44.3)	10 622 (43.1)	0.024
Platelet aggregation inhibitors	9833 (39.9)	610 742 (24.8)	0.327	9829 (39.9)	9620 (39.1)	0.017

ACE: angiotensin-converting enzyme; ECG: electrocardiogram; SD: standard deviation; Std. diff.: standardized mean difference.

### Risk of adverse cardiovascular outcomes before propensity score matching

**Figure 1.**
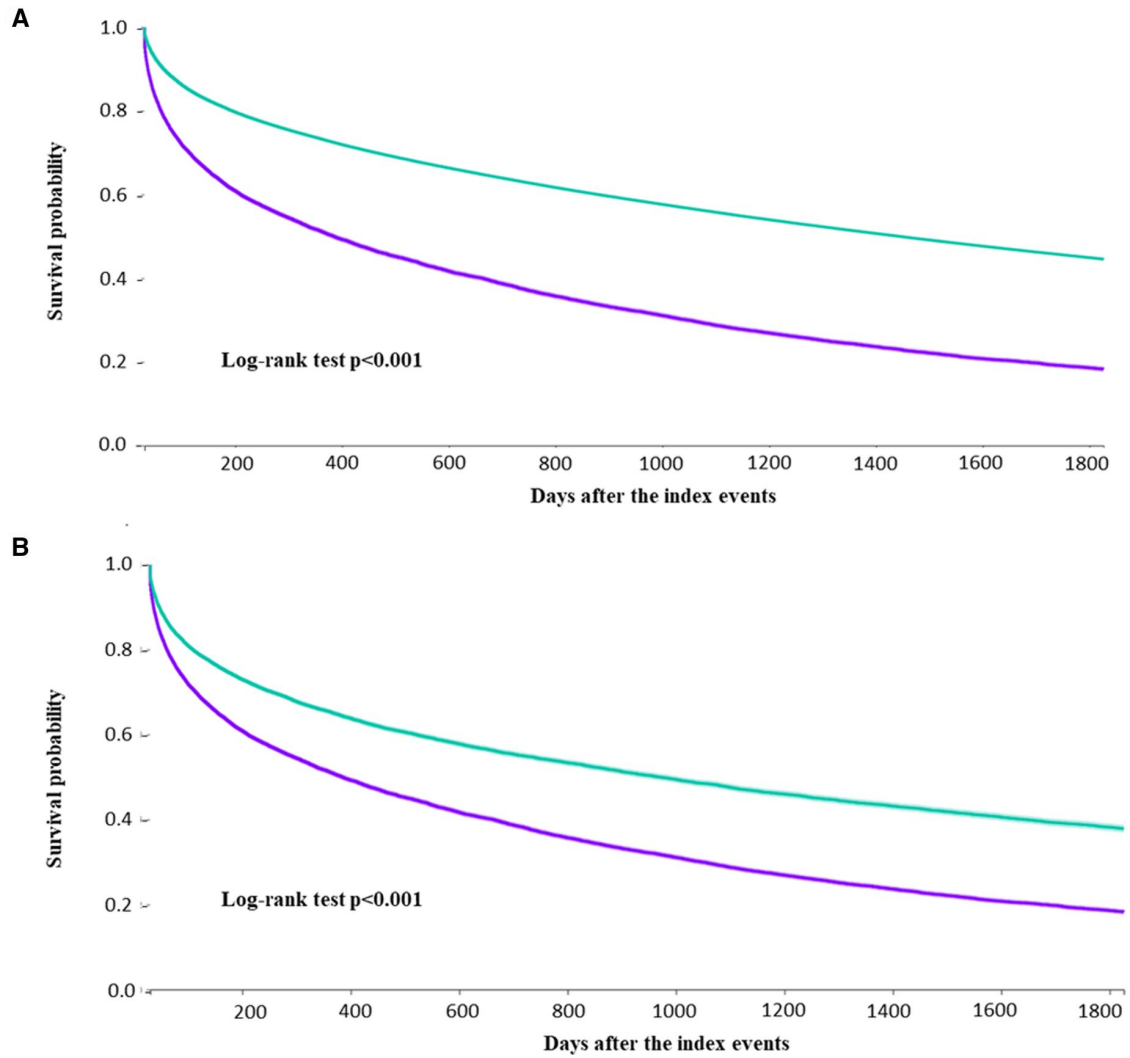
Kaplan–Meier curves for the risk of primary outcome in *socially deprived* and *non-socially deprived patients*, before (**A**) and after (**B**) propensity score matching.

The number of outcome events reported within 5 years from the index event for patients with AF socially deprived when compared to those non-deprived, as shown in Table 2 before PSM. Cox regression analysis revealed that socially deprived patients with AF showed a higher 5-year risk of composite outcome (Figure 1, Panel A), all-cause death, hospitalization, IHD, stroke, HF and severe ventricular arrhythmias, as shown in Table 2.

**Table 2.** Number and risk of adverse events before and after propensity score matching in socially deprived and non-deprived patients with atrial fibrillation

Outcome	Before PSM			After PSM		
	Socially deprived (*n* = 24 631)	Non-deprived (*n* = 2 462 092)	HR (95% CI)	Socially deprived (*n* = 24 627)	Non-deprived (*n* = 24 627)	HR (95% CI)
Composite outcome, *n* (%)	18 163 (73.8%)	1 145 707 (46.5%)	1.90 (1.87–1.93)	18 163 (73.8%)	13 473 (54.7%)	1.47 (1.44–1.50)
All-cause death, *n* (%)	2893 (11.75%)	183 310 (7.4%)	1.34 (1.28–1.39)	2893 (11.7%)	1918 (7.8%)	1.27 (1.20–1.34)
Hospitalization, *n* (%)	14 402 (58.5%)	794 359 (32.3%)	2.01 (1.98–2.04)	14 398 (58.5%)	9939 (40.4%)	1.51 (1.47–1.55)
Ischemic heart disease, *n* (%)	12 728 (51.7%)	778 981 (31.6%)	1.67 (1.64–1.70)	12 725 (35.6%)	8800 (51.2%)	1.44 (1.40–1.47)
Stroke, *n* (%)	12 728 (51.7%)	214 110 (8.7%)	2.60 (2.51–2.64)	5778 (23.5%)	2886 (11.7%)	1.88 (1.80–1.97)
Heart failure, *n* (%)	5775 (23.4%)	280 264 (11.4%)	1.91 (1.86–1.96)	5773 (23.4%)	3653 (14.8%)	1.44 (1.38–1.50)
Ventricular arrythmias, *n* (%)	2875 (11.7%)	140 045 (5.7%)	1.83 (1.76–1.90)	2875 (11.7%)	1786 (7.3%)	1.42 (1.34–1.51)

PSM: propensity score matching; HR: hazard ratio; CI: confidence interval.

### Propensity score-matched analyses

After PSM on a 1:1 ratio for the comparison between socially deprived and non-deprived patients 24 627 AF patients were included in each group. The standardized mean difference for all the variables assessed showed no substantial difference between the two groups (Table 1). The numbers of outcome events reported within 5 years from the index event for patients with AF socially deprived when compared to those non-deprived are shown in Table 2.

Cox regression analysis on the PSM cohort also found that socially deprived patients with AF had a higher 5-year risk of the composite outcome (Figure 1, Panel B), all-cause death, hospitalization, IHD, stroke, HF and severe ventricular arrhythmias.

The 5-year risk of adverse events was evaluated according to *each component of the socially deprived AF groups* compared to AF patients non-deprived after PSM 1:1. The number of patients considered were: 10 488 unemployed, 3791 with extreme poverty or with low income and 11 390 with problems related to living alone. The number of composite events in each subgroup of socially deprived patients and controls was: 8014 (76.4%) and 5658 (53.9%) in the unemployed, 8030 (70.8%) and (55.2%) in those with problems related to living alone.

The PSM HRs for the primary composite outcome were 1.54 (95% CI 1.49–1.60) for the unemployed AF patients, 1.39 (95% CI 1.31–1.47) for patients with extreme poverty or with low income and 1.42 (95% CI 1.37–1.47) for those with problems related with living alone.

## Discussion

The present study found that socially deprived patients with AF have a higher 5-year risk of all-cause death, hospitalization and cardiovascular events compared to those non-socially deprived, even after PSM. These findings were robust across all investigated domains of SDoH, including unemployment, extreme poverty, low income and having problems related to living alone.

The 34% higher risk of all-cause death we found in socially deprived AF patients from our population confirms the findings of previous studies performed in different AF cohorts. Indeed, a retrospective study performed on 4503 AF patients from the USA with a mean follow-up of 4.5 years, found that low socio-economic status was associated with a 30% increased risk of all-cause death (odds ratio 1.30, 95% CI 1.1–1.5).^23^ In a study population of 12 283 Swedish AF patients followed for 3.5 years, patients living in low socio-economic status neighborhoods showed a 49% higher risk of all-cause death compared to patients living in high socio-economic status neighborhoods (HR 1.49, 95% CI 1.13–1.96)^24^; whereas a nationwide register-based follow-up study performed in Denmark on 150 544 patients showed that after 1 year of follow-up, the risk of all-cause death in AF patients with low socio-economic status was 64% higher than those with better socio-economic conditions (HR 1.64, 0.61–0.68).^25^ Thus, compared to these results, our study suggests the presence of a wide heterogeneity of the impact of low socio-economic status in determining the risk of death in different geographical areas, which could be related to several factors such as the presence of health insurance-based health systems, different welfare policies and the presence of economic and social supports to the poorest of the populations.

The reasons behind the higher risk of mortality in socially deprived patients with AF are complex and involve the interaction among social, political and traditional risk factors. Patients with low socio-economic status have a higher chance of having health illiteracy, and thus to do not understand the importance of healthy lifestyles, including healthy diet, regular exercise and avoiding smoking; and to be less compliant with the recommended pharmacotherapies, and the required medical follow-up needed to early recognize the onset of symptoms and signs associated with cardiovascular diseases.^13^ The close relationship between SDoH and the risk of cardiovascular events we found in our study seems to confirm these hypotheses. Indeed, we identified a social gradient in cardiovascular morbidity in AF patients, where unemployment, extreme poverty, low income and having problems related to living alone were associated with a higher 5-year risk of stroke, HF, IHD and hospitalization. This confirms the findings of a Nationwide Population-Based Cohort Study performed on 317 017 Korean AF patients, where those with low socio-economic status were associated with a higher risk of emergency department visit, 30- and 90-day mortality and rehospitalization after the demission,^26^ and the higher risk of stroke (HR 1.17, 95% CI 1.05–1.30) and myocardial infarction (HR 1.18, 95% CI 0.98–1.41) described in AF patients with low income compare to those with high income, as shown in a large insurance database study on more than 300 000 AF patients from the USA.^27^

In our cohort, each SDoH component (unemployment, extreme poverty, low income and having problems related to living alone) was associated with a different effect size in determining the risk of composite outcome in AF patients, suggesting that the underlying mechanisms behind the high risk of adverse events in socially deprived patients may be heterogeneous and require future detailed evaluation.^28^ This is even more important considering the Atrial fibrillation Better Care (ABC) pathway advocated by the last international guidelines^29^^,^^30^ and the compliance which was associated with a significative reduction of the risk of hospitalization, cardiovascular events, and all-cause death in different AF populations.^31^^,^^32^ The ABC pathway is an integrated clinical approach based on three main pillars: ‘A’ avoid stroke with oral anticoagulation; ‘B’ better management of the symptoms with a patient-centered symptom-directed decisions on rate or rhythm control; and ‘C’ Cardiovascular risk factor optimization and lifestyle changes.^33^ One of the pivotal concepts of this holistic approach is that the risk of adverse events in AF patients already anticoagulated is due to the coexistence of other cardiovascular risk factors that often coexist in those patients. In light of the results of this study, SDoHs should be considered in the same way as a traditional risk factor and thus utilized for a more accurate risk stratification. Furthermore, targeted patient education and information to those who are socially deprived may influence other patient-related factors related to the health outcome (e.g. lifestyle behavior, persistence to prescribed medications and underuse of health services). Hence, SDoH could represent a future possible target for such integrated care approaches to be considered together with the classical cardiovascular risk factors, with the aim of reducing the risk of morbidity and mortality in AF patients.

### Limitations

There are several limitations to consider while interpreting the results. Healthcare organization EMR data are subject to entry errors and data gaps, and some diagnoses may be underreported, while outcomes that occurred outside the network may have not been well captured and only those with an obvious social deprivation at the hospitalization may be registered as such. This resulted in only 1% of the cohort being registered as socially deprived according to any of the three exposures and could imply some under-detection, misclassification or selection bias. Moreover, administrative data may fail to identify a relatively significant proportion of patients with AF and thus may bias estimates between SDoH and prognosis. However, it would be expected that the patients not captured by the database would be even worse off regarding both SDoH and health outcomes, meaning that what is presented in this study may just be the tip of the iceberg. The fact that low income and extreme poverty are related to an increased risk of hospitalization implies that the costs do not affect the willingness to seek hospitalization, or that the actual disparity in the need for hospitalization is even bigger than identified in the present study. Furthermore, the study is limited by the inability to stratify the analysis according to sex or ethnicity to identify possible different patterns in the social disparities in the clinical outcomes after AF among men and women as well as in different ethnic groups.

Another possible limitation of this study is the lack of statistical analysis aimed at assessing the changes in SDoH over time. SDoH are dynamic entities that should be confirmed in different time frames. We considered as baseline characteristics the information reported before the index event and cannot exclude that some of the patients considered in the socially deprived groups ameliorated their condition over time and vice versa.

## Conclusion

In patients with AF, social deprivation is associated with an increased risk of death and adverse cardiac events. There is a need for the implementation of strategies to eliminate health inequalities among AF patients.

## Supplementary Material

hcad275_Supplementary_Data

## Data Availability

The data underlying this article will be shared upon reasonable request to the corresponding author.
