# Supplementary material for: Social determinants of health and clinical outcomes among patients with atrial fibrillation: evidence from a global federated health research network
Source: QJM. 2023 Dec 7;117(5):353–9. doi: 10.1093/qjmed/hcad275 (PMC11150002; doi:10.1093/qjmed/hcad275)
Supplement: hcad275_Supplementary_Data [file hcad275_supplementary_data.zip › List of Abbrevation.docx]

List of abbreviations:

AF: Atrial fibrillation

CI: Confidence of interval

EMR: Electronic Medical Records

HR: Hazard Ratio

IHD: ischemic heart disease

HF: Heart Failure

PSM: Propensity Score Matching

SD: Standard Deviation

SDoH: Social Determinants of Health

Std. diff: Standardized mean differences
