# Supplementary material for: Social determinants of health and clinical outcomes among patients with atrial fibrillation: evidence from a global federated health research network
Source: QJM. 2023 Dec 7;117(5):353–9. doi: 10.1093/qjmed/hcad275 (PMC11150002; doi:10.1093/qjmed/hcad275)
Supplement: hcad275_Supplementary_Data [file hcad275_supplementary_data.zip › Supplementary data.docx]

**Social determinants of health and clinical outcomes among patients with atrial fibrillation. Evidence from a global federated health research network.**

Amalie H. Simoni, Tommaso Bucci, Giulio F. Romiti, Juliane Frydenlund,

Søren Paaske Johnsen, Azmil H. Abdul-Rahim, Gregory Y. H. Lip

**SUPPLEMENTARY DATA**

**Supplementary detail regarding methods**

TriNetX Database. The data are stored on the TriNetX database via a physical server at the institution’s data centre or a virtual hosted appliance. The TriNetX platform comprises of a series of these appliances connected into a federated network. This network can broadcast queries to each appliance. Results are subsequently collected and aggregated. Once the data are sent to the network, it is mapped to a standard and controlled set of clinical terminologies and undergoes a data quality assessment including ‘data cleaning’ that rejects records which do not meet the TriNetX quality standards. The TriNetX database performs internal and extensive data quality assessment with every refresh based on conformance, completeness, and plausibility (http://doi.org/10.13063/2327-9214.1244). HIPAA (Health Insurance Portability and Accountability Act) compliance of the clinical patient data is achieved using deidentification. Available data types within the network include demographics, diagnoses (represented by ICD-10-CM codes), procedures (coded in ICD-10-PCS or CPT), and measurements (coded to LOINC). The advantage of electronic health record data over insurance claim data is that both insured and uninsured patients are included. An advantage of electronic health record data over survey data is that the former represents the diagnostic rates in the population presenting to healthcare facilities. This provides an accurate account of the burden of specific diagnoses on healthcare systems. One primary limitation of relying on diagnoses is that they do not account for undiagnosed patients who might have a condition but have not yet received medical support. Another general limitation of electronic health record data is that a patient may be seen in different healthcare organizations for different components of their care. If one healthcare organization is not part of the federated network, then part of their medical records may not be available. Using a network of healthcare organizations, rather than a single site, limits this possibility but does not fully remove it. Propensity Score Matched Analyses Using logistic regression [Logistic Regression of the scikit-learn package in Python (version 3.7)], TriNetX performs a 1:1 greedy nearest neighbor matching model, with a caliper of 0.1 pooled standard deviations. To eliminate bias resulting from nearest neighbour algorithms, the orders of rows are randomized. Any baseline characteristic with a standardised mean difference between cohorts lower than 0.1 is deemed well matched (https://www.tandfonline.com/doi/full/10.1080/00273171.2011.568786).

**Supplementary Table 1**. ICD-10-CM-codes for comorbidities diagnosis.

|  | ICD-10-CM-codes |
| --- | --- |
| Arterial hypertensive disease | - I10 [primary/essential hypertension] - I11 [Hypertensive heart disease] - I12 [Hypertensive chronic kidney disease] - I13 [Hypertensive heart and chronic kidney disease] - I15 [Secondary hypertension] - I16 [Hypertensive crisis] |
| Heart Failure | - I50.1 [Left ventricular failure, unspecified] - I50.2 [Systolic (congestive) heart failure] - I50.3 [Diastolic (congestive) heart failure] - I50.4 [Combined systolic (congestive) and diastolic (congestive) heart failure] - I50.8 [Other heart failure] - I50.9 [Heart failure, unspecified] |
| Ischemic heart disease | - I20 [Angina pectoris] - I21 [Acute myocardial infarction] - I22 [Subsequent ST elevation (STEMI) and non-ST elevation (NSTEMI) myocardial infarction] - I23 [Certain current complications following ST elevation (STEMI) and non-ST elevation (NSTEMI) myocardial infarction (within the 28 days period)] - I24 [Other acute ischemic heart diseases] - I25 [Chronic ischemic heart disease] |
| Cerebrovascular disease | - I60 [Nontraumatic subarachnoid hemorrhage] - I61 [Nontraumatic intracerebral hemorrhage] - I62 [Other and unspecified nontraumatic intracranial hemorrhage] - I63 [Cerebral infarction] - I65 [Occlusion and stenosis of precerebral arteries, not resulting in cerebral infarction] - I66 [Occlusion and stenosis of cerebral arteries, not resulting in cerebral infarction] - I67 [Other cerebrovascular diseases] - I68 [Cerebrovascular disorders in diseases classified elsewhere] - I69 [Sequelae of cerebrovascular disease] |
| Diabetes mellitus | - E08 [Diabetes mellitus due to underlying condition] - E09 [Drug or chemical induced diabetes mellitus] - E10 [Type 1 diabetes mellitus] - E11 [Type 2 diabetes mellitus] - E13 [Other specified diabetes mellitus] |
| Overweight/obesity and other hyperalimentation | - E65 [Localized adiposity] - E66 [Overweight and obesity] - E67 [Other hyperalimentation] - E68 [Sequelae of hyperalimentation] |
| Chronic kidney disease | - N18.1 [Chronic kidney disease, stage 1] - N18.2 [Chronic kidney disease, stage 2] - N18.3 [Chronic kidney disease, stage 3] - N18.4 [Chronic kidney disease, stage 4] - N18.5 [Chronic kidney disease, stage 5] - N18.6 [End stage renal disease] - N18.9 [Chronic kidney disease, unspecified] |
| Neoplasms | - C00-C14 [Malignant neoplasms of lip, oral cavity and pharynx] - C15-C26 [Malignant neoplasms of digestive organs] - C30-C39 [Malignant neoplasms of respiratory and intrathoracic organs] - C40-C41 [Malignant neoplasms of bone and articular cartilage] - C43-C44 [Melanoma and other malignant neoplasms of skin] - C45-C49 [Malignant neoplasms of mesothelial and soft tissue] - C50 [Malignant neoplasms of breast] - C51-C58 [Malignant neoplasms of female genital organs] - C60-C63 [Malignant neoplasms of male genital organs] - C64-C68 [Malignant neoplasms of urinary tract] - C69-C72 [Malignant neoplasms of eye, brain and other parts of central nervous system] - C73-C75 [Malignant neoplasms of thyroid and other endocrine glands] - C76-C80 [Malignant neoplasms of ill-defined, other secondary and unspecified sites] - C7A [Malignant neuroendocrine tumors] - C7B [Secondary neuroendocrine tumors] - C81-C96 [Malignant neoplasms of lymphoid, hematopoietic and related tissue] - D00-D09 [In situ neoplasms] - D10-D36 [Benign neoplasms, except benign neuroendocrine tumors] - D37-D48 [Neoplasms of uncertain behavior, polycythemia vera and myelodysplastic syndromes] - D3A-D3A [Benign neuroendocrine tumors] - D49-D49 [Neoplasms of unspecified behavior] |
| Dyslipidemia | - E78 [Disorders of lipoprotein metabolism and other lipidemias] - E78.0 [Pure hypercholesterolemia] - E78.1 [Pure hyperglyceridemia] - E78.2 [Mixed hyperlipidemia] - E78.3 [Hyperchylomicronemia] - E78.4 [Other hyperlipidemia] - E78.5 [Hyperlipidemia, unspecified] - E78.6 [Lipoprotein deficiency] - E78.7 [Disorders of bile acid and cholesterol metabolism] - E78.8 [Other disorders of lipoprotein metabolism] - E78.9 [Disorder of lipoprotein metabolism, unspecified] |
| Pulmonary heart disease | - I26 [Pulmonary embolism] - I27 [Other pulmonary heart diseases] - I27.0 [Primary pulmonary hypertension] - I27.1 [Kyphoscoliotic heart disease] - I27.2 [Other secondary pulmonary hypertension] - I27.8 [Other specified pulmonary heart diseases] - I27.9 [Pulmonary heart disease, unspecified] - I28 [Other diseases of pulmonary vessels] |
| Chronic lower respiratory diseases | - J40 [Bronchitis, not specified as acute or chronic] - J41 [Simple and mucopurulent chronic bronchitis] - J42 [Unspecified chronic bronchitis] - J43 [Emphysema] - J44 [Other chronic obstructive pulmonary disease] - J44.0 [Chronic obstructive pulmonary disease with (acute) lower respiratory infection] - J44.1 [Chronic obstructive pulmonary disease with (acute) exacerbation] - J44.9 [Chronic obstructive pulmonary disease, unspecified] - J45 [Asthma] - J47 [Bronchiectasis] |

**Supplemental Table 2.** ICD-10-CM codes for the composite outcome.

| **Social disparity categories** | **ICD-10-CM-codes**  The composite of any of the following: |
| --- | --- |
| Ischemic heart disease | - I20 [unstable angina] - I21 [acute myocardial infarction] - I22 [subsequent ST elevation and non-ST elevation myocardial infarction] - I23 [Certain current complications following ST elevation (STEMI) and non-ST elevation (NSTEMI) myocardial infarction] - I24 [Other acute ischemic heart diseases] - I25 [Chronic ischemic heart disease] |
| Stroke | - I63 Cerebral infarction - I65 Occlusion and stenosis of precerebral arteries, not resulting in cerebral infarction - I66 Occlusion and stenosis of cerebral arteries, not resulting in cerebral infarction - I67 Other cerebrovascular diseases - I68 Cerebrovascular disorders in diseases classified elsewhere - I69 Sequelae of cerebrovascular disease |
| Acute Heart Failure | I50.21 Acute systolic (congestive) heart failure  I50.31 Acute diastolic (congestive) heart failure  I50.811 Acute right heart failure  I50.33 Acute on chronic diastolic (congestive) heart failure  I50.23 Acute on chronic systolic (congestive) heart failure  I50.43 Acute on chronic combined systolic and diastolic (congestive) heart failure  I50.813 Acute on chronic right heart faiure |
| Hospitalization | TriNetX spcific code n° 1013659 for hospital Inpatient Services |
| Severe ventricular arrhythmias | I49.9 Ventricular fibrillation or flutter  I47.2 Ventricular tachycardia |
| Any cause of death | TriNetX coded variable |
